# Protocols for Monitoring Unconventional Protein Secretion Using Luminescence and Trapping Approaches

**DOI:** 10.1002/cpz1.70326

**Published:** 2026-02-16

**Authors:** Eloïse Néel, Morgane Denus, William Fargues, Charline Gal, Camille Enjolras, Ana Boulanger, Marie‐Laure Parmentier, Julien Villeneuve

**Affiliations:** ^1^ IGF, Univ Montpellier, CNRS, Inserm Montpellier France; ^2^ These authors contributed equally to this work

**Keywords:** intracellular compartments, protein trafficking, Retention Using Selective Hooks, RUSH, secretory pathways, split luciferase, unconventional protein secretion

## Abstract

Unconventional protein secretion (UcPS) enables the export of cytosolic proteins through pathways that bypass the canonical endoplasmic reticulum–Golgi secretory route. Although increasingly recognized as essential for intercellular communication, stress responses, and tissue homeostasis, UcPS remains difficult to quantify due to low secretion efficiency, high intracellular background, and the challenge of distinguishing active secretion from passive leakage. Recent methodological advances, including NanoLuc split luciferase–based reporters and the Retention Using Selective Hooks (RUSH) system for synchronized protein transport, have improved sensitivity and temporal control of trafficking. Here, we present complementary protocols integrating these tools to provide a highly sensitive, quantitative workflow centered on a split NanoLuc (HiBiT/LgBiT) complementation assay for monitoring UcPS in mammalian cells. The Basic Protocol describes a robust luminescence‐based secretion assay, while the Support Protocols detail the generation of stable HiBiT reporter cell lines, approaches for probing UcPS mechanisms using siRNA‐mediated gene knockdown and pharmacological perturbation, and the incorporation of the RUSH system to synchronize cargo release and identify potential trafficking intermediates. Together, these protocols provide a sensitive, scalable, high‐throughput toolkit that enables analysis of UcPS mechanisms across diverse cargo proteins, cell types, and perturbations. This methodological framework allows for rigorous dissection of UcPS pathways in both physiological and disease‐relevant contexts. © 2026 The Author(s). *Current Protocols* published by Wiley Periodicals LLC.

**Basic Protocol**: Split luciferase complementation assay for quantifying UcPS in mammalian cells

**Support Protocol 1**: Generation of stable cell lines expressing HiBiT‐tagged cargo proteins for the split luciferase assay

**Support Protocol 2**: siRNA‐mediated knockdown to assess the role of candidate genes in UcPS

**Support Protocol 3**: Pharmacological perturbation of UcPS

**Support Protocol 4**: Integration of the RUSH system to synchronize UcPS

## Introduction

In eukaryotic cells, protein secretion into the extracellular space is a fundamental process essential for intercellular communication, immune responses, and tissue homeostasis. Classically, protein export occurs via the canonical endoplasmic reticulum (ER)–Golgi secretory pathway, in which proteins containing an N‐terminal signal sequence are co‐translationally translocated into the ER and subsequently trafficked through the Golgi apparatus for secretion. Over the past two decades, however, accumulating evidence has revealed that many cytosolic proteins lacking signal sequences can also be released actively into the extracellular space through unconventional protein secretion (UcPS) pathways that bypass the classical ER‐Golgi route (Chen et al., 2025; Filaquier et al., 2022; Rabouille et al., 2012; Zhang & Schekman, 2013). These pathways export a diverse range of cargos, including inflammatory cytokines, heat shock proteins, lipid chaperones, annexins, galectins, and aggregation‐prone proteins, many of which play critical roles in stress adaptation and disease pathogenesis (Lee et al., 2016; Liu et al., 2024; Villeneuve et al., 2018; Wu et al., 2023; Zhang et al., 2020).

The discovery of UcPS has significantly expanded our understanding of protein export mechanisms but also introduced new complexity. Rather than following a unified pathway, UcPS encompasses at least four mechanistically distinct types. Types I and II involve direct translocation of proteins across the plasma membrane via protein channels and ABC transporters, respectively. Type III UcPS directs cytosolic proteins through intracellular compartments such as autophagosomes, multivesicular bodies (MVBs), or lysosomes, which are then repurposed for secretion. Type IV UcPS involves ER‐to‐plasma membrane trafficking of specific transmembrane proteins, bypassing the Golgi apparatus (Chiritoiu‐Butnaru et al., 2022; Dimou & Nickel, 2018; Gee et al., 2018; Néel et al., 2024; Rabouille, 2017).

Despite major advances in identifying molecular players, quantitative analysis of UcPS remains challenging. Protein secretion levels are typically low and the extracellular fraction often represents only a minimal proportion of the total cellular pool. Consequently, standard biochemical and imaging approaches such as western blotting, immunofluorescence, and ELISA often lack the sensitivity or specificity to distinguish genuine UcPS events from passive leakage caused by cell stress or death. Immunoprecipitation is time‐consuming and low‐throughput, while mass spectrometry, though powerful, is not readily scalable for screening applications. Additionally, since Type I‐III UcPS cargos are cytosolic, live‐cell imaging approaches are confounded by high background fluorescence from the non‐secreted intracellular pool.

To overcome these limitations, we developed a luminescence‐based reporter assay (Denus et al., 2025) using the split NanoLuc luciferase system (HiBit/LgBiT; Dixon et al., 2016). This assay provides a sensitive, cost‐effective, and quantitative means to measure UcPS of cytosolic proteins. It is readily scalable to high‐throughput screening and can be combined with Retention Using Selective Hooks (RUSH) synchronization (Boncompain et al., 2012) to temporally control cargo release and track intracellular trafficking, particularly within Type III pathways.

Here, we describe a set of complementary protocols for implementing this cell‐based assay to study a wide range of UcPS cargos across various cell types and physiological or pathological conditions. The Basic Protocol details the split‐luciferase assay and data analysis. Support Protocol 1 describes the generation of stable HiBiT reporter cell lines. Support Protocols 2 and 3 outline the application of this assay for analysis of genetic and pharmacological perturbations of secretion, respectively. Support Protocol 4 details the integration of the RUSH system to synchronize and monitor cargo trafficking. Together, these protocols provide a robust and versatile toolkit for mechanistic investigation of UcPS. The combined approaches enable quantitative, highly sensitive, and high‐throughput analysis of UcPS dynamics under physiological and pathological conditions.

*NOTE*: All solutions and equipment coming into contact with cells must be sterile, and proper sterile technique should be used accordingly. Work should be carried out in a sterile laminar flow hood.

## SPLIT LUCIFERASE COMPLEMENTATION ASSAY FOR QUANTIFYING UcPS IN MAMMALIAN CELLS

**Figure 1:**
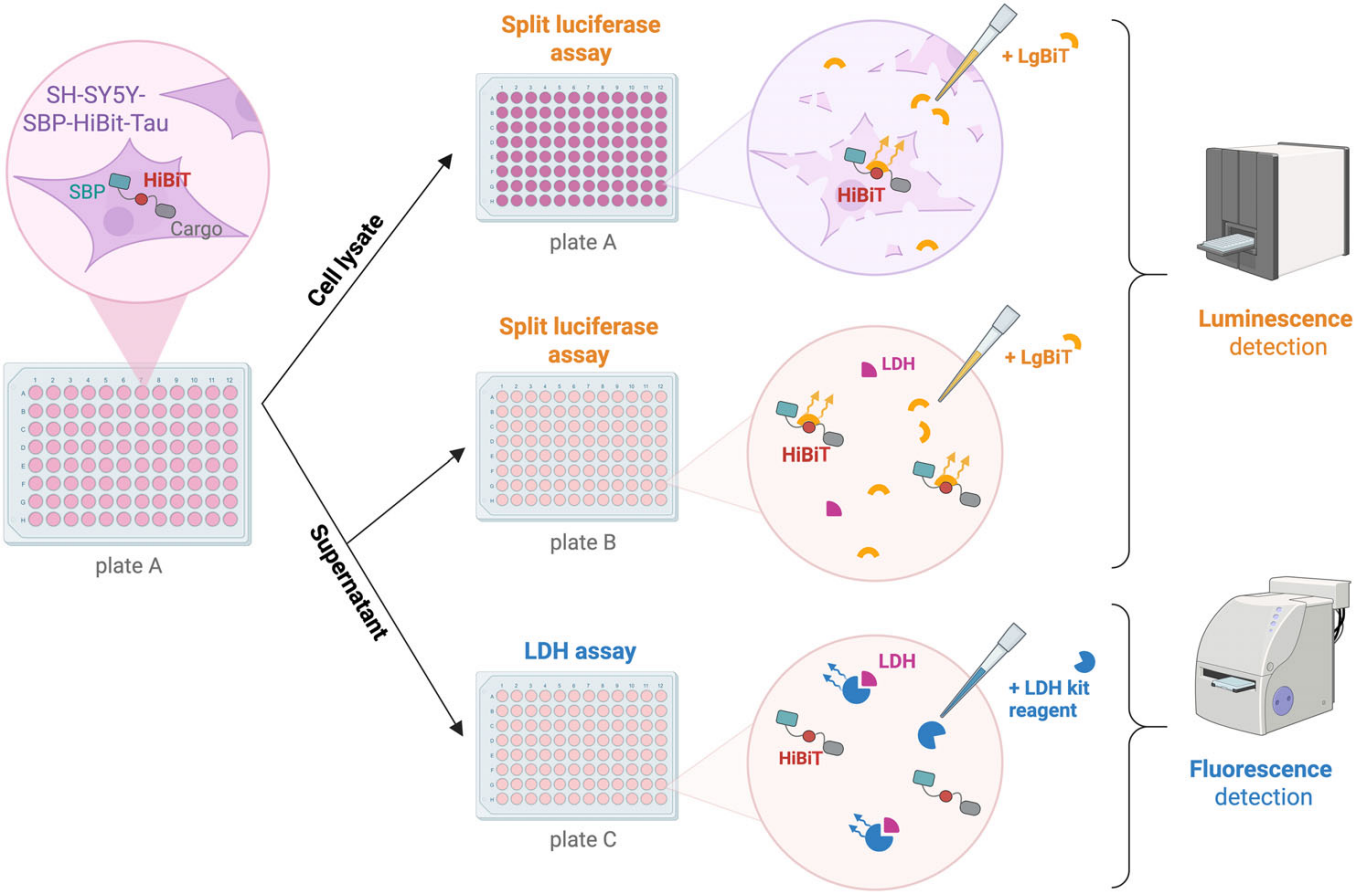
Workflow for split luciferase and LDH assays. SH‐SY5Y cells stably expressing a HiBiT‐tagged construct are seeded into 96‐well plates (plate A). After washing, cells are incubated for 0‐12 hr, depending on the desired kinetic resolution (one plate is used for time point). At each time point, the conditioned medium is collected and distributed to two additional plates: plate B for luminescence measurement and plate C for LDH activity measurement. To detect intracellular versus extracellular luminescence (plates A and B, respectively), LgBiT protein is added to reconstitute luciferase activity and luminescence is measured using a plate reader. LDH activity is determined from plate C using a standard LDH assay. Figure generated with BioRender.

This protocol describes a quantitative and reproducible method for measuring UcPS in mammalian cells using a split NanoLuc luciferase (HiBiT/LgBiT) system. The overall workflow of the assay is illustrated in Figure 1. The cargo protein of interest (e.g., Tau) is fused at its N terminus to the HiBiT peptide (11 amino acids). Upon addition of the complementary LgBiT fragment, active NanoLuc luciferase is reconstituted, producing a luminescent signal proportional to the amount of HiBiT‐tagged protein. Luminescence is measured in both cell supernatants (secreted pool) and cell lysates (intracellular pool) to calculate secretion ratios. Parallel assessment of lactate dehydrogenase (LDH) activity serves as a control for cytosolic leakage or cell lysis. The method is demonstrated here using SH‐SY5Y human neuroblastoma cells stably expressing HiBiT‐tagged Tau and HiBiT‐tagged GFP as a negative control, but can be readily adapted to other cell types and cargo proteins. Kinetic experiments are typically conducted by collecting samples at regular intervals over a 12‐hr culture period to assess secretion dynamics over time.

### Materials

SH‐SY5Y cells stably expressing HiBiT‐tagged Tau or GFP (see Support Protocol 1)
Complete growth medium for SH‐SY5Y cells (see recipe)
Dulbecco's phosphate‐buffered saline (DPBS) without Ca^2+^/Mg^2+^ (Sigma‐Aldrich, cat. no. D8537)
Nano‐Glo HiBiT Lytic Detection System (Promega, cat. no. N3040), including lytic buffer, lytic substrate, and LgBiT protein
Nano‐Glo HiBiT Extracellular Detection System (Promega, cat. no. N2421), including extracellular buffer, extracellular substrate, and LgBiT protein
LDH activity assay kit (Thermo Fisher Scientific, cat. no. C20300)
Antibodies for validation by western blotting:
  Anti‐GFP (Abcam, cat. no. ab6556)
  Anti‐SBP (Santa Cruz Technology, cat. no. sc‐101595)
  Anti‐HiBit (Promega, cat. no. CS2006A01)

Black, clear‐bottom, 96‐well tissue culture plates (PerkinElmer, cat. no. 6005182)
37°C, 5% CO_2_ incubator
200‐µl multichannel pipette and reagent reservoir
Plate reader (Tecan's Spark 20M multimode microplate reader)
GraphPad Prism software (www.graphpad.com) or equivalent

#### Culture cells

1 In a sterile environment, plate SH‐SY5Y cells expressing HiBiT‐tagged construct in black, clear‐bottom, 96‐well plates at 200 µl/well in complete growth medium. Grow cells in a 37°C, 5% CO_2_ incubator to 60%‐70% confluence.
  Use one line with the desired HiBiT‐tagged cargo (e.g., Tau) and one with HiBiT‐tagged GFP as a control. For kinetic experiments, prepare one plate per time point to avoid repeated manipulation of cells intended for later time points. For each time point and experimental conditions, use six replicate wells, and avoid edge wells, which often exhibit higher variability than interior wells.
2 Gently rinse cells three times with complete growth medium and immediately return the plate to the incubator.
  This is considered t = 0 of the kinetic experiment.
  Perform washes slowly using a multichannel pipette to minimize mechanical stress. Use prewarmed medium rather than DPBS to prevent osmotic and/or metabolic stress.
3 Incubate for 2‐12 hr (depending on the desired kinetic resolution) in a final volume of 200 µl complete medium per well.

#### Collect samples

4 At each time point, remove the corresponding culture plate (designated plate A) from the incubator. Collect 50 µl of conditioned medium per well and transfer to a new plate (plate B) for luminescence measurement (Fig. 1). Collect an additional 50 µl and transfer to another plate (plate C) for LDH activity measurement. Include three extra wells in plates B and C containing only fresh medium as an extracellular background control. Keep plates B and C on ice (for no longer than 1‐2 hr before assays).
  Maintain identical well layout between plates A‐C. Avoid disturbing the cell layer with the pipette tip during medium collection to prevent potential cytosolic leakage from damaged cells.
5 Remove remaining medium (∼100 µl) from plate A by inverting and gently blotting on absorbent paper. Wash cells gently three times with 200 µl DPBS.
6 After washing, add 50 µl DPBS to each well and keep on ice (for no longer than 1‐2 hr).
  Include three wells in plate A with only DPBS as a negative control for intracellular background luminescence.

#### Measure luminescence

Intracellular luminescence is measured in plate A using the Nano‐Glo HiBiT Lytic Detection System, which permeabilizes the cells to reveal the intracellular pool of reporter protein. Extracellular luminescence is measured in plate B using the Nano‐Glo HiBiT Extracellular Detection System. Measure luminescence from plates A and B immediately after each time point.

7 Just before use, prepare the appropriate detection mix (all volumes are per well).
*For plate A*:
    48.5 µl lytic buffer
    0.5 µl LgBiT protein
    1 µl lytic substrate
*For plate B*:
    48.5 µl extracellular buffer
    0.5 µl LgBiT protein
    1 µl extracellular substrate
      Detection mixes should be prepared fresh before use and dispensed rapidly using a multichannel pipette to minimize time‐based variation. The prepared mixes can be kept on ice, protected from light, for up to 2‐4 hr without significant loss of activity.
8 Add 50 µl of the appropriate mix to each well of the appropriate plate (A and B).
9 Incubate plate B at room temperature with gentle agitation on an orbital shaker (300‐500 rpm) for 5‐10 min. For plate A, incubate for at least 10 min to ensure complete permeabilization.
10 Measure luminescence using a plate reader (integration time 0.5‐2 s, gain auto‐adjusted).

#### Perform LDH assay

11 Measure LDH activity from plate C according to the kit manufacturer's instructions.
  Elevated LDH activity indicates loss of cell membrane integrity and potential nonspecific leakage.

#### Analyze and normalize data

To correct for variation in expression levels, cell number, and background signal, secretion data are normalized using a secretion index.

**Figure 2:**
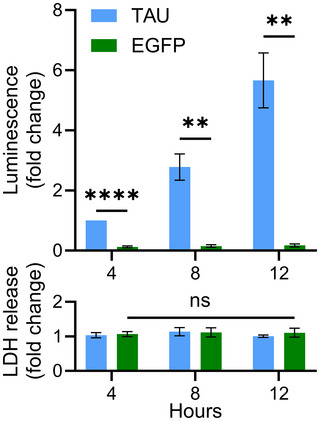
Results of split luciferase assay for protein secretion. SH‐SY5Y cells expressing HiBiT‐Tau incubated in complete medium and sampled at 4‐hr intervals over a 12‐hr time course. The ratio of medium to lysate luminescence was quantified and values for each condition were normalized to *t* = 4 hr. As a control, the assay was performed in SH‐SY5Y cells expressing EGFP fused to HiBiT and a streptavidin tag. Data are mean ± SD of three biological replicates as analyzed by two‐way ANOVA (**p ≤ 0.01, ****p < 0.0001 vs. EGFP) followed by Tukey's multiple comparison test. The bottom graph shows LDH release (mean ± SD of three biological replicates; two‐way ANOVA followed by Tukey's multiple comparison test). The specificity of the assay was validated by the observed luciferase activity, which increased over time for Tau but not for EGFP, and by the absence of a difference in LDH release among all conditions.

12 Subtract background luminescence values (from cell‐free wells) from the values of all samples and timepoints.
13 Calculate the ratio of extracellular (supernatant) to intracellular (lysate) luminescence for each condition:
$$\mathrm{secretion}\;\mathrm{index}\;=\;\frac{\;L_{\sup\;}-\;L_{\sup,\;\mathrm{background}\;}}{L_{\mathrm{lys}\;}-L_{\mathrm{lys},\;\mathrm{background}}}$$
14 Normalize the secretion index values to the designated control condition, such as non‐targeting siRNA (see Support Protocol 2) or vehicle‐treated cells (see Support Protocol 3), which is assigned a value of 1.0.
15 For kinetic experiments, plot normalized secretion index value (also called fold‐change luminescence) over time (e.g., 2‐12 hr) to visualize secretion dynamics.
  Example data are shown in Figure 2. For bona fide UcPS cargos such as Tau, extracellular luminescence should increase progressively over time with minimal LDH release, confirming active secretion rather than passive leakage due to cell damage.

## Support Protocol 1 GENERATION OF STABLE CELL LINES EXPRESSING HiBiT‐TAGGED CARGO PROTEINS FOR THE SPLIT LUCIFERASE ASSAY

Stable expression of HiBiT‐tagged cargo proteins ensures consistent and reproducible quantification of UcPS using the split NanoLuc luciferase assay described in the Basic Protocol. This protocol outlines the generation of SH‐SY5Y cell lines stably expressing HiBiT‐tagged reporter constructs. As demonstrated previously (Denus et al., 2025), the approach can be adapted for diverse cargos secreted via the conventional ER‐Golgi pathway (e.g., tumor necrosis factor α [TNFα]) or the UcPS pathways (e.g., fibroblast growth factor‐2 [FGF2], interleukin‐1β [IL1β], galectin‐3 [Gal3], α‐synuclein [αSNC], superoxide dismutase‐1 [SOD1], and Tau). HiBiT‐tagged GFP‐expressing cells serve as a negative control for secretion. Here, we describe generation of SH‐SY5Y cells stably expressing HiBiT‐tagged Tau as a model UcPS cargo, including antibiotic selection and fluorescence‐activated cell sorting (FACS). Comparing secretion profiles of multiple UcPS cargos with GFP secretion, combined with kinetic analyses and LDH activity assays, enables discrimination between active, UcPS‐mediated secretion and nonspecific protein release resulting from cell lysis or stress.

### Materials

**Figure 3:**
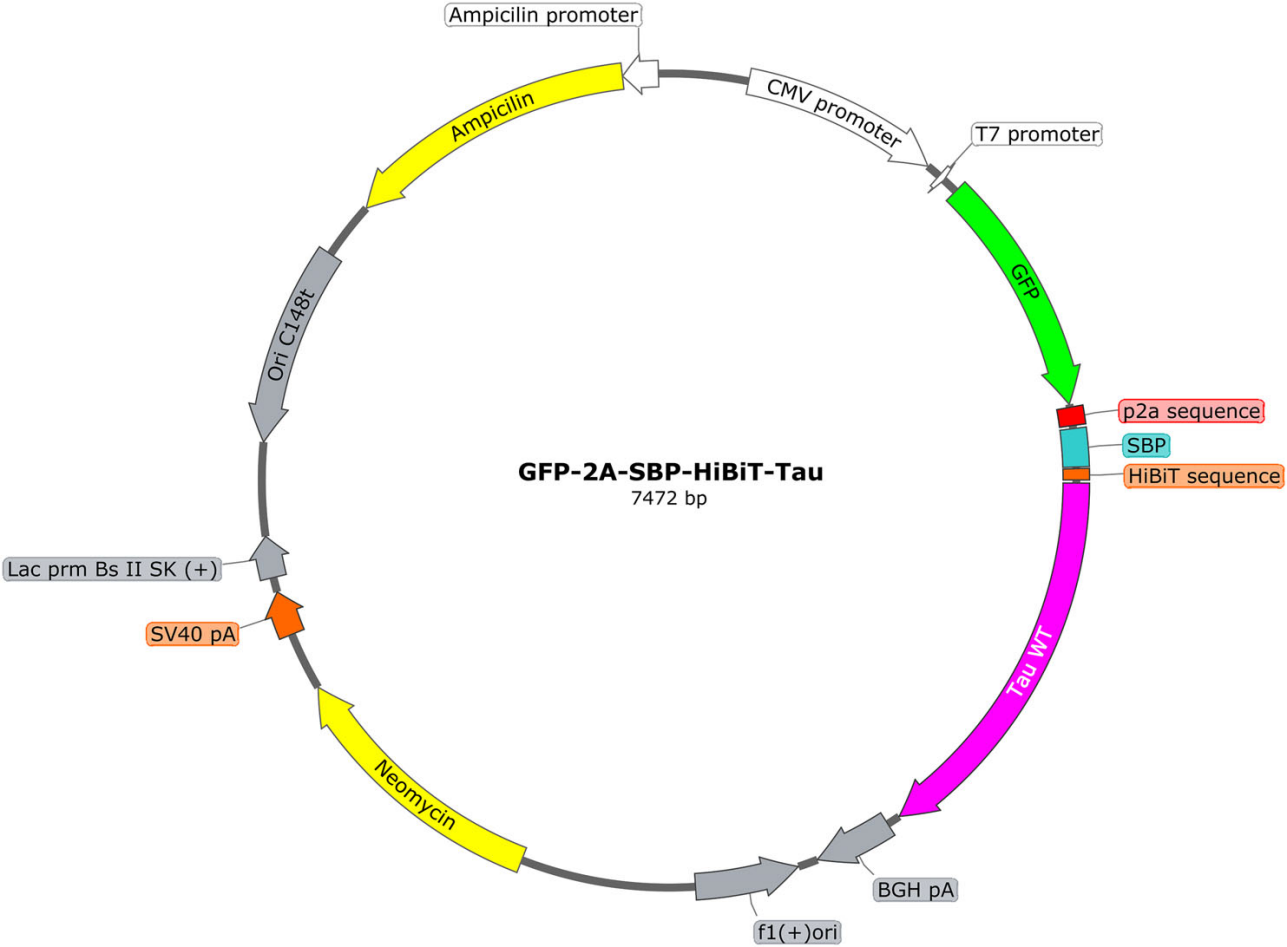
Vector map for the pcDNA3.1‐GFP‐2A‐SBP‐HiBiT‐Tau construct used as an example in Support Protocol 1 to generate an SH‐SY5Y cell line stably expressing Tau. It encodes the reporter SBP‐HiBiT‐Tau co‐expressed with GFP, which serves as a marker of stable transfection, enabling fluorescence‐based sorting. A self‐cleaving 2A peptide enables co‐translational expression of GFP and SBP‐HiBiT‐Tau from a single plasmid. Similar constructs can be generated for a variety of cargo proteins secreted via either the conventional ER‐Golgi‐dependent pathway or UcPS pathways. Notably, a pcDNA3.1‐GFP‐2A‐SBP‐HiBiT‐GFP construct serves as a negative control for secretion. Vector map generated using SnapGene.

SH‐SY5Y cells (ATCC CRL‐2266)
Complete growth medium (see recipe) with P/S, without P/S, and with P/S plus 20% FBS
Opti‐MEM reduced‐serum medium (Gibco, cat. no. 31985‐062)
TransIT‐2020 transfection reagent (Euromedex, cat. no. MIR5400)
100 ng/ml expression plasmid pcDNA3.1‐GFP‐2A‐SBP‐HiBit‐Tau (Thermo Fisher Scientific; Fig. 3) containing cargo of interest (SBP‐HiBiT‐Tau), fluorescent marker (GFP), and antibiotic resistance cassette (neomycin/G‐418)
Dulbecco's phosphate‐buffered saline (DPBS) without Ca^2+^/Mg^2+^ (Sigma‐Aldrich, cat. no. D8537)
G‐418 sulfate (geneticin; Sigma, cat. no. 4727878001)
0.05% trypsin‐EDTA (Gibco, cat. no. 25300‐054)

10‐cm tissue culture dishes (Corning, cat. no. 353003)
37°C, 5% CO_2_ incubator
Fluorescence microscope
40‐µm cell strainer (Fisher Scientific, cat. no. 22363547)
Cell sorter (FACS Discover S8)

#### Culture and transfect cells

1 In a sterile environment, culture SH‐SY5Y cells in 10 ml complete growth medium in a 10‐cm tissue culture dish. Grow cells in a 37°C, 5% CO_2_ incubator to 60%‐70% confluence.
2 Prepare transfection reagent‐DNA complex (for one 10‐cm dish):
  1000 µl Opti‐MEM reduced‐serum medium
  10 µg 100 ng/ml plasmid DNA
  40 µl TransIT‐2020 transfection reagent
  Prepare fresh for each experiment. Mix gently and incubate for 15‐20 min at room temperature before adding to cells.
3 Wash cells twice with 10 ml DPBS, then add 9 ml antibiotic‐free growth medium (no P/S).
  The absence of antibiotics improves transfection efficiency.
4 Add the transfection complex dropwise to the dish, swirling gently to distribute evenly. Return to the incubator for 6 hr.
5 Replace medium with complete growth medium (with P/S) and continue incubating for 48 hr.
6 Replace medium with complete growth medium plus 600 µg/ml G‐418 to select for transfected cells.
  Prior to selection, perform a kill curve to determine the minimal G‐418 concentration that eliminates >95% of untransfected cells within 5 days. For SH‐SY5Y cells, 600 µg/ml G‐418 is typically sufficient.
7 Maintain cells under G‐418 selection for at least 10 days, replacing the medium every 2‐3 days to remove dead cells.
8 Monitor cultures by fluorescence microscopy for emergence of GFP‐positive, G‐418 resistant cells.
  After 10 days, only cells with stable integration of the construct should remain.
  If cell survival is low, extend selection time or consider lentiviral transduction for improved integration efficiency.

#### Sort and expand transfected cells

9 After antibiotic selection, wash cells three times with 10 ml DPBS.
10 Detach cells by treating with 5 ml of 0.05% trypsin‐EDTA for 5 min at 37°C.
11 Add 5 ml complete growth medium to inactivate trypsin and centrifuge at 150 × g for 5 min.
12 Discard supernatant and resuspend cell pellet in 1 ml Opti‐MEM containing 20% FBS.
  The higher serum concentration (20% FBS instead of 10%) improves cell viability during FACS sorting.
13 Pass the suspension through a 40‐µm cell strainer to remove cell aggregates. Keep suspension on ice.
14 Sort cells based on GFP fluorescence signal intensity and collect sorted cells into complete growth medium containing 20% FBS.
  Set gate for background fluorescence using untransfected SH‐SY5Y cells as a negative control. To establish a polyclonal population, collect bulk GFP‐positive cells into a 15‐ml tube containing complete medium. To generate monoclonal lines, sort single cells into 96‐well plates.
15 Expand sorted cells gradually in complete growth medium supplemented with a low‐dose selection antibiotic (300 µg/ml G‐418, half the selection concentration) to maintain selective pressure.
  During expansion, validate transgene expression by western blotting using antibodies against GFP, SBP, and HiBit to confirm expression and expected molecular weight.
  Aliquots of validated cells can be frozen in liquid nitrogen for long‐term storage.

#### Perform split luciferase and LDH assays

16 Assess secretion using the split NanoLuc luciferase and LDH assays (see Basic Protocol).
  Kinetic experiments are recommended to characterize secretion dynamics.

## Support Protocol 2 siRNA‐MEDIATED KNOCKDOWN TO ASSESS THE ROLE OF CANDIDATE GENES IN UcPS

The split luciferase assay is a sensitive and highly adaptable method suitable for multiple experimental formats including 96‐well plates, making it a powerful tool for targeted genetic screening (Denus et al., 2025). This protocol describes the use of short interfering RNA (siRNA)–mediated gene silencing in SH‐SY5Y cells stably expressing reporter fusion proteins, followed by quantification of UcPS using the split luciferase assay. Specifically, SH‐SY5Y cells expressing either SBP‐HiBit‐tagged Tau (an unconventionally secreted cargo) or TNFα (a signal sequence–containing cargo) are transiently transfected with siRNAs targeting genes of interest. As an example, this protocol uses siRNAs against VAMP7 (identified as a regulator of Tau UcPS; Lopez et al., 2024) and SCFD1 (an essential component of ER‐to‐Golgi transport for conventional secretion). Non‐targeting siRNA serves as a negative control. The protocol provides detailed procedures for siRNA transfection and subsequent quantitative analysis of reporter secretion using the split NanoLuc luciferase assay as described in the Basic Protocol.

A two‐step siRNA transfection strategy is employed in which cells are first transfected while still in suspension and then re‐transfected after they have adhered. This strategy enhances knockdown efficiency, particularly for genes with slow turnover or in cells with moderate transfection susceptibility.

### Materials

SH‐SY5Y cells stably expressing SBP‐HiBiT‐Tau or SBP‐HiBit‐TNFα (see Support Protocol 1)
Complete growth medium (see recipe) with and without P/S
Dulbecco's phosphate‐buffered saline (DPBS) without Ca^2+^/Mg^2+^ (Sigma‐Aldrich, cat. no. D8537)
0.05% trypsin‐EDTA (Gibco, cat. no. 25300‐054)
Lipofectamine 2000 transfection reagent (Invitrogen, cat. no. 11668019)
Opti‐MEM reduced‐serum medium (Gibco, cat. no. 31985‐062)
siRNAs (typically 20 µM stock; ON‐TARGETplus, Horizon Discovery; see Table 1)

10‐cm tissue culture dishes (Corning, cat. no. 353003)
37°C, 5% CO_2_ incubator
Sterile microcentrifuge tubes (Eppendorf, cat. no. 15625367)

**Table 1:** ON‐TARGETplus siRNA pools for Gene Silencing (from Horizon Discovery)

Pool	Catalog number	Sequences
Non‐targeting pool	D‐001810‐10‐50	5′‐UGGUUUACAUGUCGACUAA‐3′
		5′‐UGGUUUACAUGUUGUGUGA‐3′
		5′‐UGGUUUACAUGUUUUCUGA‐3′
		5′‐UGGUUUACAUGUUUUCCUA‐3′
Human SCFD1 siRNA smartpool	L‐010943‐01‐0005	5′‐AAGCAUUGGUGCACGAUGU‐3′
		5′‐GACAAGAAACUUCGAGAAA‐3′
		5′‐GUGCCAGGAUCUUCGAAAU‐3′
		5′‐GAUAUCACAGACACGGAAA‐3′
Human VAMP7 siRNA smartpool	L‐020864‐00‐0005	5′‐GUACUCACAUGGCAAUUAU‐3′
		5′‐GAACGUUCCCGAGCCUUUA‐3′
		5′‐CGAGUUCUCAAGUGUCUUA‐3′
		5′‐GCCAAGACAGGAUUGUAUA‐3′

#### Culture and harvest cells

1 In a sterile environment, culture SH‐SY5Y cells carrying the desired cargo in 10 ml complete growth medium in a 10‐cm culture dish. Grow cells in a 37°C, 5% CO_2_ incubator to 80%‐90% confluence.
2 Wash cells three times with 10 ml DPBS.
3 Detach cells by incubating with 5 ml of 0.05% trypsin‐EDTA for 5 min at 37°C.
4 Neutralize trypsin by adding 5 ml antibiotic‐free medium (no P/S) and centrifuge at 150 × g for 5 min.
  Antibiotics can reduce transfection efficiency and must be omitted from this and subsequent steps.
5 Discard supernatant and resuspend cell pellet gently in antibiotic‐free medium.
6 Seed 5 × 10³ cells/well into 96‐well plates in 160 µl total volume.

#### Transfect cells in suspension

7 In a sterile microcentrifuge tube, dilute 0.2 µl Lipofectamine 2000 in 20 µl Opti‐MEM. Incubate for 5 min at room temperature.
8 In a separate tube, dilute 20 µM siRNA in 20 µl Opti‐MEM to reach the desired final concentration (typically 20‐50 nM in the final transfection volume).
9 Combine the diluted siRNA and Lipofectamine 2000 solutions (1:1 volume ratio) and mix gently. Incubate 15‐20 min at room temperature to allow complex formation.
10 Add the 40 µl siRNA‐Lipofectamine mixture to the 160 µl cell suspension.
  The final concentrations of transfection mix are 0.1% Lipofectamine, 20‐50 nM siRNA, and 20% Opti‐MEM.
11 Mix gently by pipetting and immediately plate into 96‐well plates.
12 Incubate 24 hr at 37°C, 5% CO_2_.

#### Re‐transfect cells after attachment

13 Replace medium with 160 µl fresh antibiotic‐free medium.
14 Prepare fresh siRNA‐Lipofectamine complex as above (see steps 7‐9).
  Be sure to maintain identical siRNA and Lipofectamine 2000 concentrations.
15 Add 40 µl freshly prepared complex dropwise to each well containing adherent cells and rock the plate gently to distribute the complex evenly.
16 Return plate to the incubator and culture for an additional 48 hr.
  For each targeted gene, knockdown efficiency must be validated, e.g., by western blotting.
17 Wash cells and collect samples as described (see Basic Protocol, steps 2‐6).

#### Perform split luciferase and LDH assays

**Figure 4:**
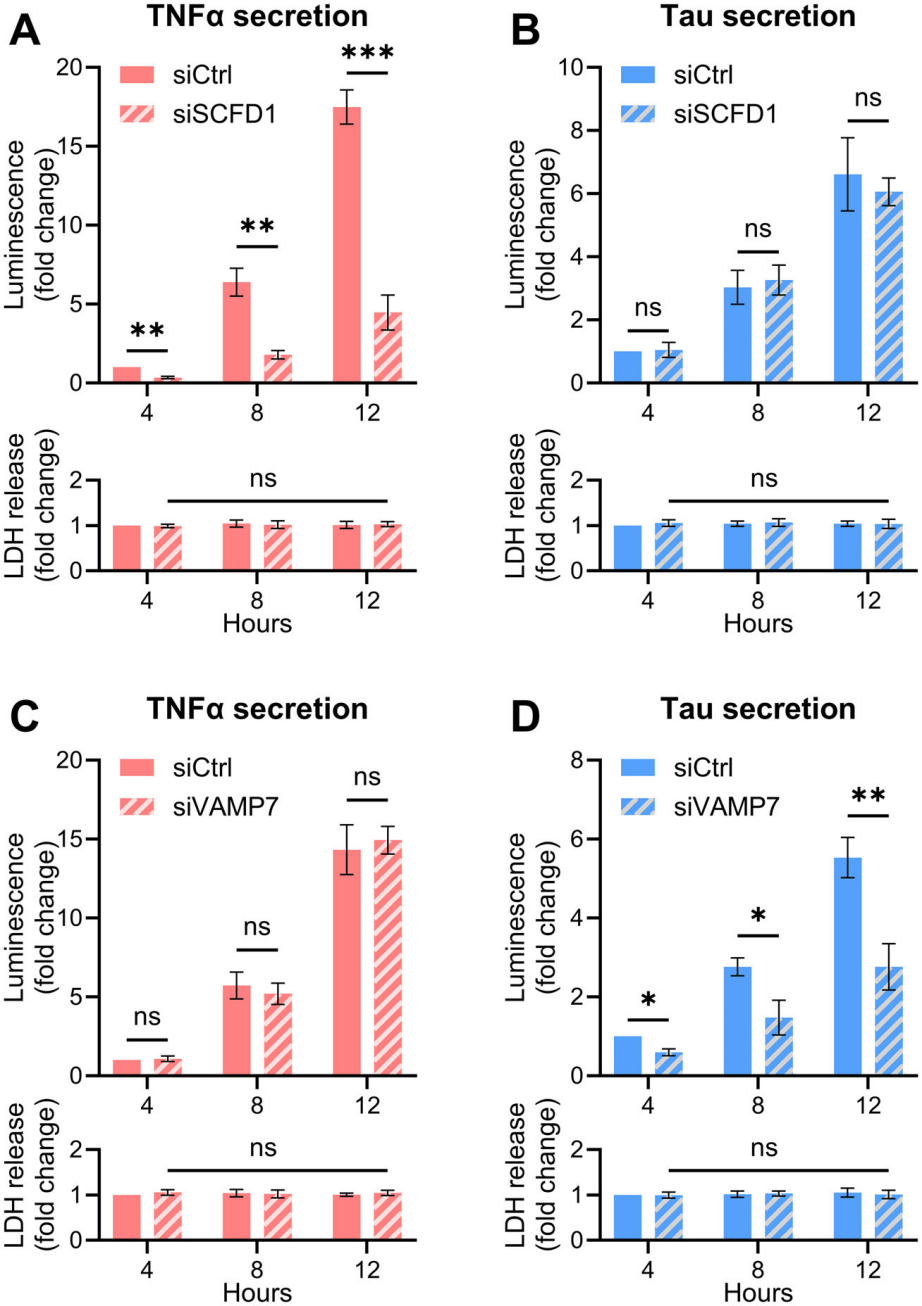
Results of split luciferase assay following siRNA‐mediated knockdown of targeted genes. SH‐SY5Y cells stably expressing HiBiT‐tagged cargo protein TNFα (A, C) or Tau (B, D) were transfected with control siRNA or siRNA targeting SCFD1 (A, B) or VAMP7 (C, D). Cells were incubated in complete medium and sampled at 4‐hr intervals over 12 hr. The ratio of medium to lysate luminescence was quantified and the values for each condition were normalized to the control siRNA at *t* = 4 hr. Data are mean ± SD of three biological replicates (*p ≤ 0.05, **p ≤ 0.01, ***p < 0.001 vs. ctrl siRNA) as analyzed by two‐way ANOVA followed by Tukey's multiple comparison test. The bottom graphs show LDH release from each fraction (mean ± SD of three biological replicates; two‐way ANOVA followed by Tukey's multiple comparison test).

18 Assess secretion using the split NanoLuc luciferase and LDH assays (see Basic Protocol). Express results relative to the non‐targeting siRNA control to determine the effect of each gene knockdown on UcPS activity.
  Kinetic experiments are recommended to characterize secretion dynamics. For example results, see Figure 4.

## Support Protocol 3 PHARMACOLOGICAL PERTURBATION OF UcPS

Pharmacological modulation of the secretory pathway provides a rapid and reversible approach to distinguish UcPS from canonical ER‐Golgi‐dependent secretion. Because the split luciferase assay is a sensitive and highly adaptable method suitable for multiple experimental formats including 96‐well plates, it is also a powerful tool for pharmacological screening (Liu et al., 2024). This protocol describes the use of small‐molecule inhibitors targeting key trafficking and degradation steps to probe mechanistic aspects of UcPS. The assay is performed using SH‐SY5Y cells lines stably expressing HiBiT‐tagged Tau (an unconventionally secreted cargo) or TNFα (a signal sequence–containing cargo). Cells are incubated with brefeldin A, which blocks ER‐to‐Golgi trafficking, and bafilomycin A1, a V‐ATPase inhibitor that has been shown to enhance Tau UcPS (Lopez et al., 2024). This protocol outlines a detailed step‐by‐step procedure for drug treatment, followed by quantitative assessment of secreted reporter activity using the split luciferase assay.

### Materials

SH‐SY5Y cells stably expressing SBP‐HiBiT‐Tau or SBP‐HiBit‐TNFα (see Support Protocol 1)
Complete growth medium (see recipe)
Dimethyl sulfoxide (DMSO; Sigma‐Aldrich, cat. no. D8418)
Brefeldin A (BFA; Sigma‐Aldrich, cat. no. B6542)
Bafilomycin A1 (BafA1; Enzo, cat. no. BML‐CM110)

Black, clear‐bottom, 96‐well tissue culture plates (PerkinElmer, cat. no. 6005182)
37°C, 5% CO_2_ incubator

**Figure 5:**
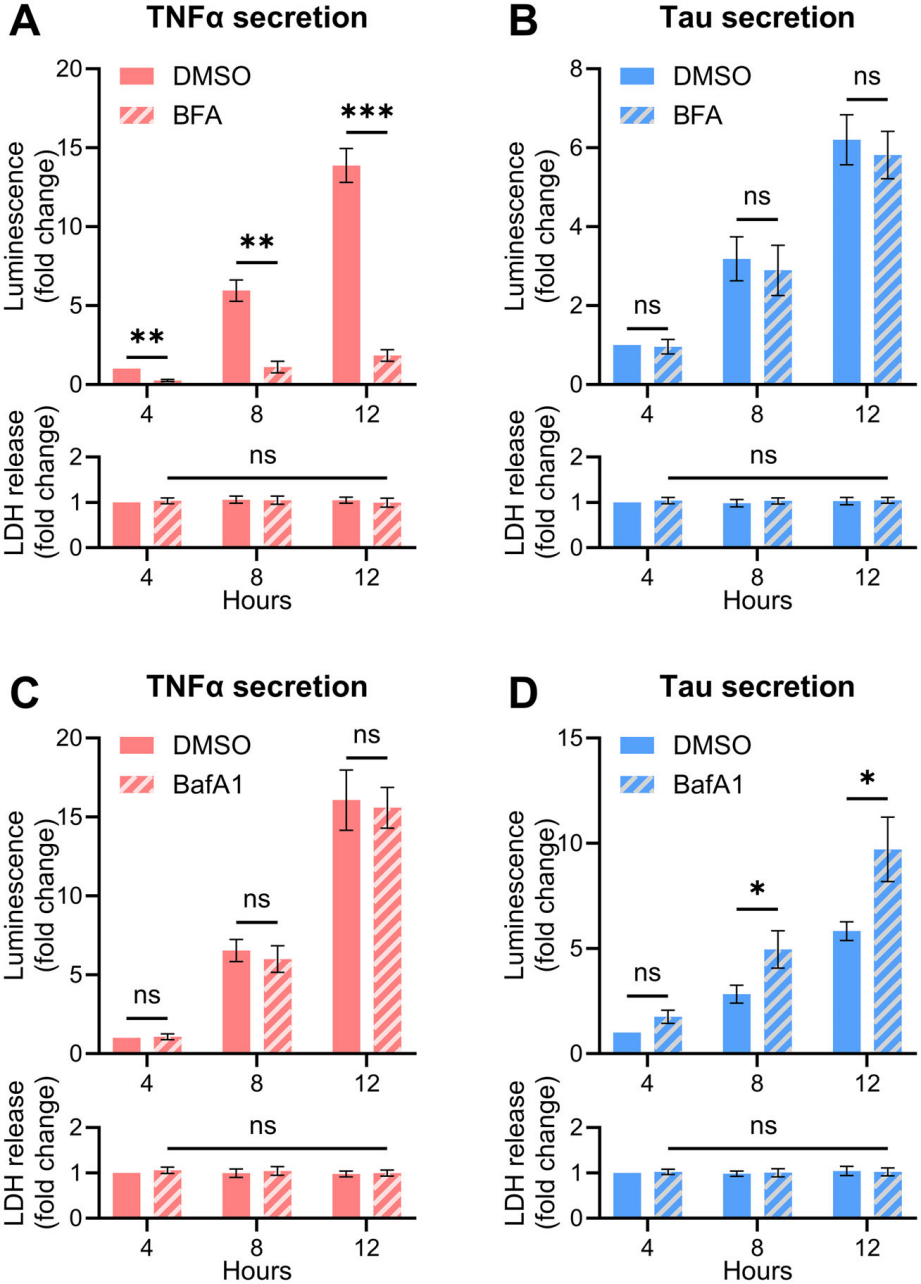
Results of split luciferase assay after pharmacological modulation of the secretory pathway. SH‐SY5Y cells stably expressing HiBiT‐tagged cargo protein TNFα (A, C) or Tau (B, D) were incubated in complete medium in the presence of DMSO, 500 ng/ml BFA, or 100 nM BafA1 for 12 hr with sampling at 4‐hr intervals. The ratio of medium to lysate luminescence was quantified and the values for each condition were normalized to the control sample (DMSO) at *t* = 4 hr. Data are mean ± SD of three biological replicates (*p ≤ 0.05, **p ≤ 0.01, ***p < 0.001 vs. DMSO) as analyzed by two‐way ANOVA followed by Tukey's multiple comparison test. The bottom graphs show LDH release from each fraction (mean ± SD of three biological replicates; two‐way ANOVA followed by Tukey's multiple comparison test.

1 In a sterile environment, culture SH‐SY5Y cells in black, clear‐bottom, 96‐well plates at 200 µl/well in complete growth medium. Grow cells in a 37°C, 5% CO_2_ incubator to 60%‐70% confluence.
2 Gently rinse cells three times with complete growth medium.
3 Immediately add DMSO (vehicle control), 500 ng/ml BFA, or 100 nM BafA1 and return the plate to the incubator.
  This time is considered t = 0 of the kinetic experiment.
4 Incubate plates for 2, 4, 6, or 12 hr (depending on the desired kinetic resolution) in a final volume of 200 µl/well.
5 Perform split luciferase and LDH assays as described (see Basic Protocol).
  For data analysis, normalize secretion indices to DMSO‐treated cells. For example results, see Figure 5.

## Support Protocol 4 INTEGRATION OF THE RUSH SYSTEM TO SYNCHRONIZE UcPS

**Figure 6:**
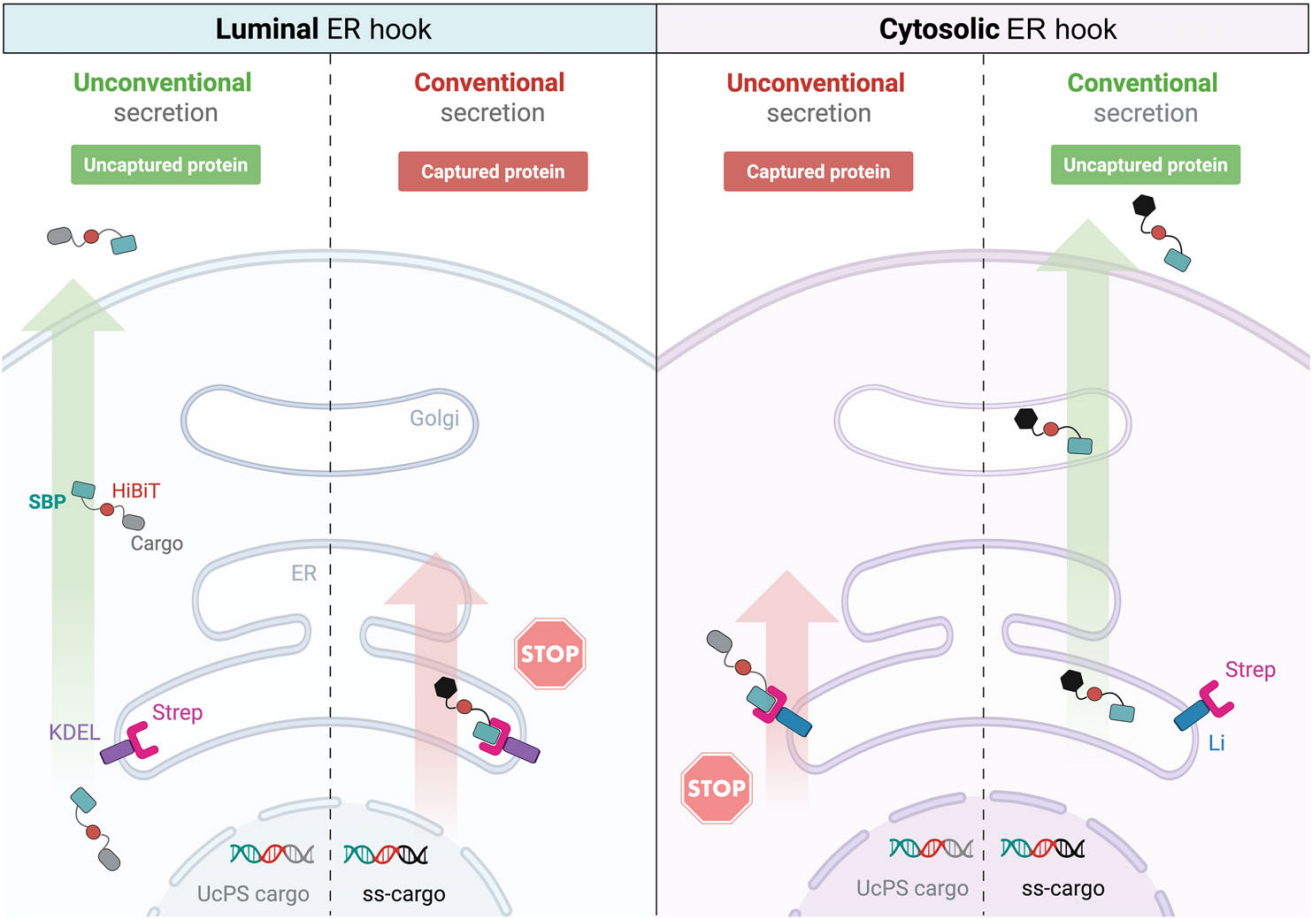
Schematic representation of the different configurations used for the RUSH system. In SH‐SY5Y cells, expression of an ER hook with streptavidin (Strep) facing the ER lumen (left) is expected to have no effect on UcPS but prevent conventional secretion of cargo containing a signal sequence (ss) via the ER‐Golgi pathway. In contrast, expression of a hook with Strep facing the cytosol (right) is expected to inhibit UcPS but have no effect on secretion of ss‐containing cargo protein. Importantly, the inhibition of cargo protein secretion by the Strep‐SBP interaction is prevented by the addition of biotin. Figure generated with BioRender.

The Retention Using Selective Hooks (RUSH) system (Boncompain et al., 2012) allows the synchronization of protein trafficking by retaining a reporter cargo in a specific intracellular compartment until release is triggered by adding biotin. In the context of UcPS, the RUSH system enables controlled release of cytosolic cargos and mapping of their intracellular trafficking routes, particularly for Type III UcPS. This protocol describes the integration of RUSH with the HiBiT split luciferase assay for temporal and mechanistic analysis of UcPS cargo trafficking and secretion in SH‐SY5Y cells. The cargo protein of interest is fused to a streptavidin‐binding peptide (SBP) tag and co‐expressed with a “hook” protein that contains a streptavidin (Strep) domain anchored to a specific organelle (Fig. 6). In the absence of biotin, the SBP‐cargo is retained via the SBP‐Strep interaction. Addition of biotin competitively disrupts this interaction, leading to synchronous release and trafficking of the cargo and allowing monitoring of trafficking and secretion in real time. The use of hooks with Strep localized to the ER lumen versus the cytosol enables the separation of UcPS from conventional secretion via the ER‐Golgi (Fig. 6).

### Materials

HEK293T cells (ATCC CRL‐3216)
Complete growth medium for HEK293 cells (see recipe) with and without P/S
Opti‐MEM reduced‐serum medium (Gibco, cat. no. 31985‐062)
TransIT‐2020 transfection reagent (Mirus, cat. no. MIR5400)
Plasmids:
  pCDH‐Str‐KDEL (ER lumen‐facing hook; Addgene, cat. no.65307)
  pCDH‐Str‐Ii (ER cytosolic‐facing hook; Addgene, cat. no. 65313)
  psPAX lentiviral packaging plasmid (Addgene, cat. no. 12260)
  pMD2.G VSV‐G envelope plasmid (Addgene, cat. no. 12259)
Dulbecco's phosphate‐buffered saline (DPBS) without Ca^2+^/Mg^2+^ (Sigma‐Aldrich, cat. no. D8537)
SH‐SY5Y cells stably expressing SBP‐HiBiT‐Tau, SBP‐HiBit‐TNFα, or SBP‐HiBit‐IL1β (see Support Protocol 1)
Complete growth medium for SH‐SY5Y cells (see recipe) with and without P/S
Polybrene (Millipore, cat. no. TR‐1003‐G)
Puromycin (Sigma‐Aldrich, cat. no. P9620)
Anti‐streptavidin antibody (Santa Cruz Biotechnology, cat. no. sc‐52236) for validation of hook construct
Biotin (Sigma‐Aldrich, cat. no. B4639)

10‐cm tissue culture dishes (Corning, cat. no. 353003)
37°C, 5% CO_2_ incubator
0.45‐µm low protein‐binding filter (VWR, cat. no. 28145‐479)

#### Culture cells (day 1)

1 In a sterile environment, plate HEK293T cells in 10‐cm dishes in complete growth medium. Grow cells in a 37°C, 5% CO_2_ incubator to reach ∼50% confluence the next day.

#### Prepare lentivirus (day 2)

2 Prepare the following transfection reagent–DNA complex for each 10‐cm dish:
  1 ml Opti‐MEM
  10 µg hook construct (pCDH‐Str‐KDEL or pCDH‐Str‐Ii)
  7.5 µg psPAX2
  2.5 µg pMD2.G
  40 µl TransIT‐2020 reagent
  Mix gently and incubate 15 min at room temperature.
  Prepare fresh transfection complex for each experiment.
3 Wash HEK293T cells twice with DPBS.
4 Add 9 ml antibiotic‐free medium (no P/S).
  The absence of antibiotics improves transfection efficiency.
5 Add transfection complex dropwise to the dish, swirling gently to distribute evenly. Incubate at 37°C, 5% CO_2_ for 24 hr.
6 Replace medium with fresh antibiotic‐free medium and incubate for another 24 hr.
7 Collect supernatant (containing virus) and filter through 0.45‐µm low protein‐binding membrane.
8 Concentrate by ultracentrifugation at 17,000 × *g* for 1.5 hr at 4°C. Resuspend virus in 1 ml DPBS.
  Virus can be stored at −80°C for a maximum of 6‐12 months. Avoid repeated freeze‐thaw cycles.

#### Transduce SH‐SY5Y cells for stable expression of RUSH hooks

9 Plate ∼1 × 10⁶ SH‐SY5Y cells stably expressing SBP‐HiBiT‐Tau, SBP‐HiBit‐TNFα, or SBP‐HiBit‐IL1β in 10‐cm dishes in antibiotic‐free medium (for SH‐SY5Y cells, no P/S) containing 8 µg/ml polybrene.
10 Add 15 µl concentrated virus and incubate for 24 hr.
11 Replace with fresh antibiotic‐free medium and incubate another 24 hr.
12 Add 2 µg/ml puromycin to select for transduced cells.
  Prior to selection, perform a kill curve to determine the minimal puromycin concentration that eliminates >95% of untransduced cells within 5 days. For SH‐SY5Y cells, 2 µg/ml is typically sufficient.
13 Maintain cells under puromycin selection for at least 5 days, replacing medium every 2‐3 days to remove dead cells.
14 Expand cells gradually in complete growth medium (with P/S) with low‐dose antibiotic selection (1 µg/ml puromycin, half the selection concentration) to maintain selective pressure.
  Validate hook expression via western blotting and immunofluorescence using an antibody against streptavidin to confirm expected molecular weight expression and subcellular localization.
15 Freeze aliquots of validated cells in liquid nitrogen for long‐term storage.

#### Synchronize cargo release and perform split luciferase assay

**Figure 7:**
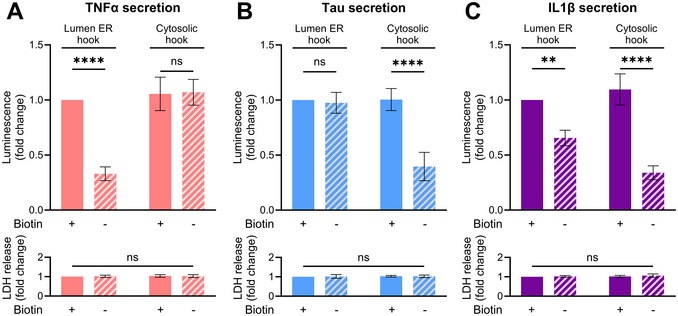
The RUSH system reveals the role of conventional secretory pathway compartments in UcPS of IL1β. Split luciferase assays were performed in SH‐SY5Y cells expressing ER hooks facing either the ER lumen or the cytosol and a cargo protein fused to the HiBit sequence and streptavidin (Strep) tag. Cargo proteins included TNFα (A), Tau (B), and IL1β (C). Cells were incubated for 12 hr in complete medium in the presence or absence of 40 µM biotin. The ratio of medium to lysate luminescence was quantified and values for each condition were normalized to the control sample (luminal ER hook, treated with biotin). Data are mean ± SD of three biological replicates (**p ≤ 0.01, ****p < 0.0001 vs. lumenal hook + biotin) as analyzed by two‐way ANOVA followed by Tukey's multiple comparison test. The bottom graphs show LDH release from each fraction (mean ± SD of three biological replicates; two‐way ANOVA followed by Tukey's multiple comparison test).

16 Culture SH‐SY5Y cells expressing ER‐luminal or ER‐cytosolic hook and SBP‐HiBiT cargos (TNFα, Tau, and IL1β) in black, clear‐bottom, 96‐well plates in 200 µl/well complete growth medium. Grow cells to 60%‐70% confluence.
17 Gently rinse cells three times with complete growth medium.
18 Immediately incubate in the presence or absence of 30 µM biotin and return to the incubator.
  This time is defined as t = 0.
19 Incubate cells for 12 hr. Maintain a 200‐µl volume complete medium throughout the incubation period.
20 Perform split luciferase and LDH assays as described (see Basic Protocol).
  Normalize secretion values to the corresponding signal obtained from cells expressing the ER‐luminal hook in the presence of biotin, which serves as the baseline for full cargo release. Example results are shown in Figure 7.

## Reagents and Solutions

### Complete growth medium for HEK293T cells

89 ml DMEM (Sigma‐Aldrich, cat. no. D6429)
10 ml heat‐inactivated fetal bovine serum (FBS, Gibco, cat. no.10270‐106)
1 ml penicillin‐streptomycin (P/S; Gibco, cat. no. 15140‐122)
Mix thoroughly
Store up to 2 weeks at 4°C
Warm to 37°C before use

### Complete growth medium for SH‐SY5Y cells

88 ml DMEM/F12 (Sigma‐Aldrich, cat. no. D8437)
10 ml heat‐inactivated fetal bovine serum (FBS, Gibco, cat. no.10270‐106)
1 ml penicillin‐streptomycin (P/S; Gibco, cat. no. 15140‐122)
1 ml MEM non‐essential amino acids (100×, Sigma‐Aldrich, cat. no. M7145)
Mix thoroughly
Store up to 2 weeks at 4°C
Warm to 37°C before use

## COMMENTARY

### Critical Parameters

#### Secretion assay

Upon receipt, all components of the Nano‐Glo HiBiT Lytic and Extracellular Detection Systems should be stored at –20°C. The required volume of Nano‐Glo HiBiT detection reagent needed to perform the desired experiments should be calculated before starting the assay. This usually constitutes a volume equal to the total amount of medium in the wells plus any extra required for dispensing. Both lytic and extracellular detection reagents must be prepared fresh immediately before use. During the assay, after the desired incubation period (typically kinetics from 0 to 12 hr), all samples (both cells and cell culture supernatants) should be placed on ice to minimize potential proteolysis.

#### Generation of stable cell lines

For optimal transfection efficiency, over‐confluence should be avoided. Cells should be 60%‐70% confluent at the time of transfection. During antibiotic selection and expansion, continuous G‐418 selection pressure should be maintained to prevent the loss of transgene expression and ensure stability of the engineered cell line.

#### Assessing secretion by perturbation

For genetic or pharmacological perturbation of UcPS, appropriate internal controls should always be included to distinguish a specific effect on UcPS from nonspecific cellular response or passive release caused by cell damage. Recommended controls include DMSO (vehicle) for pharmacological treatments, non‐targeting control siRNA for siRNA‐mediated knockdown experiments, a conventional secretion cargo (e.g., TNFα‐HiBiT), and GFP‐only control lines. Appropriate time‐course (kinetic) analyses should always be incorporated. In addition, cytotoxicity assays (such as LDH release) are essential for accurate interpretation. These controls and measures provide confidence that observed changes in extracellular luminescence reflect modified UcPS rather than global changes in secretion, transcription, or cell viability and integrity.

#### RUSH synchronization

Biotin concentration should be optimized for each cell type to achieve robust cargo release with minimal cytotoxicity. Typically, 20‐50 µM is a good range. Expression of both hook and cargo constructs should be confirmed by western blotting or fluorescence microscopy prior to performing functional assays.

### Troubleshooting

Problems may arise when monitoring UcPS using the split NanoLuc luciferase and the RUSH systems following these protocols. Tables 2, 3, 4, 5, 6 summarize the most frequently encountered issues, outline possible causes, and provide recommended solutions to facilitate successful execution.

**Table 2:** Troubleshooting for Split Luciferase Assay

Problem	Possible cause	Recommended solution
Low extracellular luminescence signal	Low expression of HiBiT‐tagged cargo	Confirm construct integrity; verify expression by western blotting; ensure stable line is maintained under selective pressure
	Insufficient secretion during incubation	Extend incubation time; verify that medium volume is correct (200 µl/well)
High extracellular signal with elevated LDH activity	Cell lysis due to mechanical stress during washes	Perform aspiration slowly; pipette against wall of well; avoid touching cell monolayer
	Osmotic stress from DPBS washes	Replace DPBS with prewarmed complete medium as recommended in protocol
Inconsistent luminescence across wells	Edge effects	Avoid using outer wells
	Uneven cell seeding density	Resuspend cells thoroughly before plating; check for homogeneous attachment after 2‐4 hr
Low intracellular luminescence signal	Incomplete cell permeabilization	Increase incubation with lytic detection mix to 10‐15 min; agitate gently on an orbital shaker
Signal saturation on plate reader	Very high expression of HiBiT cargo or prolonged incubation	Lower integration time; use automatic gain adjustment; dilute samples 2‐5× in DPBS to confirm linearity
No time‐dependent increase in extracellular signal	Cargo is not secreted via UcPS	Confirm that Tau or other model cargo behaves as expected; test positive UcPS controls
	Incubation too short or cell density too low	Extend time points (6‐12 hr); ensure 60%‐70% starting confluency
Unexpectedly low secretion index	High intracellular background or low extracellular signal	Reassess background subtraction; ensure all controls are included (medium‐only, DPBS‐only)

**Table 3:** Troubleshooting for Generation of Stable Cell Lines Expressing HiBiT‐tagged Cargo

Problem	Possible cause	Recommended solution
Low transfection efficiency	Cells too confluent or antibiotics present during transfection	Transfect at 60%‐70% confluency; remove antibiotics from medium during complex formation and transfection
	Suboptimal DNA/TransIT ratio	Confirm DNA purity (*A*_260_/*A*_280_ = 1.8‐2.0); optimize DNA/reagent ratio (typically 1 µg DNA to 3 µl reagent)
High cell death immediately after G‐418 addition	G‐418 concentration too high	Perform kill curve before selection; reduce to minimal effective concentration (typically 400‐600 µg/ml for SH‐SY5Y)
	Transfection stress	Allow cells to recover 24‐48 hr after transfection before adding G‐418; ensure cells are healthy before selection
Cells survive G‐418 but show weak or no GFP expression	Overgrowth of non‐expressing cells	Increase selection stringency; extend selection period to 10‐14 days
Very low number of surviving cells post‐selection	Harsh selection or poor plasmid integration	Reduce G‐418 dose by 25%; extend recovery before selection; consider using lentiviral transduction
Cells die or perform poorly during FACS	Shear stress or insufficient serum protection	Use 20% FBS during sorting; keep cells on ice; pass suspension through 40‐µm strainer to remove aggregates
	Prolonged sorting time	Sort in smaller batches; minimize time outside incubator
Weak fluorescence signal during sorting	Low expression of GFP marker	Verify correct GFP fusion or 2A peptide function; adjust gating to include low‐expressing but bona fide transgene‐positive cells
Cells lose expression after expansion	Loss of selective pressure	Maintain G‐418 at 50% of selection dose (i.e., 300 µg/ml) during routine culture

**Table 4:** Troubleshooting for siRNA‐mediated Gene Knockdown of UcPS

Problem	Possible cause	Recommended solution
Low knockdown efficiency	siRNA concentration too low	Increase final siRNA concentration to 30‐50 nM; perform concentration titration
	Suboptimal siRNA‐lipid complex formation	Ensure Lipofectamine‐siRNA incubation time is 15‐20 min; do not vortex
	High mRNA turnover or high protein stability	Increase transfection frequency (two‐step protocol already improves this); extend incubation to 72 hr; verify knockdown by western blotting
High cell death after transfection	Lipofectamine toxicity	Reduce Lipofectamine by 20%‐40%; increase Opti‐MEM dilution; verify cell health before transfection
	siRNA off‐target toxicity	Verify phenotype with ≥2 independent siRNAs; reduce siRNA concentration to 10‐20 nM
Cells detach or fail to adhere after first transfection	Transfection in suspension stresses cells	Ensure gentle pipetting; use prewarmed medium; avoid centrifugation forces >150 × *g*
	Low serum concentration	Maintain FBS at 10% during plating; avoid serum‐free incubation
High extracellular luminescence with elevated LDH	siRNA‐induced cytotoxicity	Reduce siRNA dose; test alternative siRNAs; shorten incubation to 48 hr

**Table 5:** Troubleshooting for Pharmacological Perturbation of UcPS

Problem	Possible cause	Recommended solution
Drug shows no measurable effect on secretion	Incorrect drug concentration or expired stock	Prepare fresh stock solutions; titrate across a 0.1‐5× range
	Drug precipitates or has poor solubility in medium	Warm DMSO stocks to room temperature before dilution; vortex gently; ensure final DMSO concentration ≤0.1%‐0.2%
	Insufficient incubation time	Extend incubation to 6‐12 hr
Cells exhibit high toxicity after drug treatment	Drug concentration too high	Perform dose‐response viability curve
Increased extracellular luminescence accompanied by high LDH	Drug‐induced cell permeabilization or stress	Confirm LDH; exclude wells with LDH >3× control; reduce drug concentration or exposure time

**Table 6:** Troubleshooting for Using the RUSH System to Synchronize UcPS

Problem	Possible cause	Recommended solution
Low or no expression of RUSH hook constructs after lentiviral transduction	Low viral titer or inefficient concentration during ultracentrifugation	Verify ultracentrifuge speed (17,000 × *g*, 1.5 hr, 4°C); increase initial virus volume
	Incorrect MOI or low transduction efficiency	Increase virus volume; add polybrene (8 µg/ml); spinoculate at 800 × *g* for 1 hr to improve transduction
	Puromycin selection too stringent or too weak	Perform a kill curve before selection; adjust puromycin to eliminate >95% of nontransduced cells in 3‐5 days
Hook protein mislocalization	Overexpression causes misfolding or ER stress	Reduce lentiviral MOI
Cargo not retained before biotin addition	Insufficient hook expression relative to SBP‐cargo	Increase hook expression (higher MOI)
	Weak streptavidin‐SBP affinity due to construct orientation	Confirm correct SBP orientation; test SBP‐N vs. SBP‐C terminus
No cargo release after biotin addition	Biotin degraded or concentration too low	Prepare fresh 30‐40 µM biotin solution; avoid repeated freeze‐thaw cycles; protect from light
Asynchronous or slow cargo release kinetics	High confluence reduces trafficking dynamics	Seed cells to 60%‐70% confluence; avoid >90% confluence at *t* = 0
High extracellular HiBiT signal prior to biotin addition	Leakage due to cell stress	Verify LDH; discard wells with LDH >2× control
Cell toxicity after biotin addition	High biotin concentration	Reduce to 10‐20 µM; titrate dose in SH‐SY5Y cells

### Statistical Analysis

All data should be reported as mean ± SD and derived from a minimum of three independent biological replicates, each measured in at least triplicate technical wells. Statistical analyses may be performed using two‐way ANOVA followed by Tukey's multiple comparison test. Significant difference is defined as *p* < 0.05. GraphPad Prism is recommended for graphical representation and statistical analyses as it provides standardized figure formats and reproducible workflows.

### Understanding Results

As described in the Basic Protocol, kinetic experiments performed using human SH‐SY5Y neuroblastoma cells stably expressing either SBP‐HiBiT‐Tau or SBP‐HiBiT‐GFP (negative control) reveal a clear time‐dependent increase in Tau UcPS (Fig. 2). In contrast, GFP secretion does not increase over time, demonstrating the specificity of the assay. Importantly, the increase in extracellular luminescence is not due to cytosolic leakage, as extracellular LDH activity remains unchanged. The possibility to compare multiple UcPS cargos against a GFP control, combined with kinetic measurements and the absence of LDH release, underscores the assay's sensitivity and specificity in distinguishing active UcPS from nonspecific protein release due to cell lysis or stress.

As detailed in Support Protocols 2 and 3, the combination of genetic perturbations (siRNA) and pharmacological inhibition enables systematic interrogation of the secretory route for specific cargo proteins. As an example, depletion of SCFD1, an essential factor for ER‐to‐Golgi transport of signal sequence–containing proteins, reduces TNFα secretion (Fig. 4A), consistent with its trafficking through the conventional ER‐Golgi pathway. In contrast, SCFD1 knockdown does not affect Tau secretion (Fig. 4B), in line with its classification as a UcPS cargo. Conversely, knockdown of VAMP7, previously identified as a regulator of Tau UcPS via lysosomal exocytosis (Lopez et al., 2024), decreases Tau secretion without altering TNFα secretion (Fig. 4C,D). Pharmacological perturbation further validates these pathway assignments. As expected, treatment with brefeldin A, an inhibitor of protein transport through the ER‐Golgi membranes, reduces TNFα secretion but does not affect UcPS cargo (Fig. 5A,B). Conversely, treatment with bafilomycin A1, a V‐ATPase inhibitor previously shown to enhance Tau UcPS (Lopez et al., 2024), significantly increases Tau secretion while leaving TNFα release unchanged (Fig. 5C,D). Under all experimental conditions tested, extracellular LDH levels remain stable (Fig. 5), confirming that increases in extracellular luminescence are not caused by membrane damage or cytosolic leakage.

Combined with the RUSH system, this assay can be used to map the intracellular compartments that mediate UcPS. In this approach, an SBP‐tagged cargo reversibly binds a streptavidin‐tagged hook localized to a defined organelle. Addition of biotin disrupts the streptavidin‐SBP interaction, releasing the cargo. As described in Support Protocol 4, SH‐SY5Y cells stably expressing HiBiT‐SBP‐TNFα, ‐Tau, or ‐IL1β were transduced with lentivirus constructs encoding hooks targeted to the ER lumen (Strep‐KDEL) or to the cytosol‐facing side of the ER membrane (Ii‐Strep). With the luminal hook, TNFα, a signal sequence–containing cargo, was efficiently trapped and its secretion restored upon addition of biotin (Fig. 7A), as expected. In contrast, TNFα secretion was unaffected by the cytosol‐facing hook. Application of the same trapping strategies to UcPS cargos revealed distinct behaviors. Tau secretion was insensitive to the luminal hook but was blocked by the cytosol‐oriented hook, with biotin reversing this inhibition (Fig. 7B), indicating that Tau secretion does not involve the ER‐ERGIC‐Golgi axis. IL1β showed a similar response to the cytosol‐facing hook; however, the luminal hook partially reduced IL1β secretion, suggesting that a subset of IL1β may access compartments associated with the conventional secretory pathway (Fig. 7C). This observation aligns with recent evidence that IL1β secretion can occur via the TMED10‐channeled UcPS (THU) pathway, in which TMED10 oligomerization facilitates UcPS cargo translocation into the ERGIC (Zhang et al., 2020; Zheng et al., 2026). Together, these results demonstrate that integrating NanoLuc Binary Technology with the RUSH system provides a powerful strategy to identify intermediate compartments used during UcPS.

### Time Considerations

In the secretion assay, the secretion incubation takes 2‐12 hr, depending on the desired kinetic resolution. Sample processing and analysis take ∼1.5 hr.

The complete workflow for generating stable cell lines typically requires 3‐5 weeks, including transfection, antibiotic selection, FACS sorting, cell expansion, and validation.

siRNA‐mediated gene knockdown takes a total of 4‐5 days, including cell plating and transfection (1 day), incubation to achieve maximal knockdown (2‐3 days), and sample processing and analysis (1 day).

Pharmacological perturbation experiments takes a total of 2 days, with 1 day for cell plating and drug treatment and 1 day for sample processing and analysis.

Experiments involving RUSH synchronization take a total of 2‐3 weeks, including lentiviral transduction, stable expression of RUSH hooks, and sample processing and analysis.

### Author Contributions

**E. Néel**: Conceptualization; formal analysis; methodology; writing—original draft. **M. Denus**: Conceptualization; formal analysis; methodology; writing—original draft. **W. Fargues**: Conceptualization; formal analysis; methodology; writing—original draft. **C. Gal**: Methodology; writing—original draft. **C. Enjolras**: Methodology; writing—original draft. **A. Boulanger**: Methodology; writing—original draft. **M.‐L. Parmentier**: Conceptualization; writing—original draft; supervision. **J. Villeneuve**: Conceptualization; funding acquisition, writing—original draft; writing—review and editing; supervision.

### Conflict of Interest

The authors declare no competing financial interests.

## Data Availability

Data, tools, and materials that support these protocols are available from the corresponding author, Julien Villeneuve (*julien.villeneuve@igf.cnrs.fr*), upon reasonable request.
